# State-specific disruptions of dynamic functional connectivity in young migraine without aura: a hidden Markov model approach

**DOI:** 10.3389/fnins.2026.1756997

**Published:** 2026-03-18

**Authors:** ChunYang Xu, Songhua Zhan, WenLi Tan, Jian-Ming Cheng, ShuHui Gong, ShiXian Lu, Hui Wang, Fei Liu, ShanShan Jiang, Wu Wang, YuChan Yang

**Affiliations:** 1 Department of Radiology, Longhua Hospital, Shanghai University of Traditional Chinese Medicine, Shanghai, China; 2 Department of Radiology, Shuguang Hospital, Shanghai University of Traditional Chinese Medicine, Shanghai, China; 3 Department of Acupuncture and Tuina, Shanghai University of Traditional Chinese Medicine, Shanghai, China

**Keywords:** brain states, default mode network, dynamic functional connectivity, hidden Markov model, migraine, resting-state fMRI

## Abstract

**Background:**

Migraine is a common neurological disorder involving network-level dysfunction. Increasing evidence suggests that migraine involves network-level dysfunction and is associated with altered resting-state functional connectivity. Traditional static functional connectivity analyses are limited in capturing the temporal dynamics of large-scale brain networks. The Hidden Markov Model (HMM) provides an advanced analytical framework to identify discrete, recurrent brain states and characterize their temporal properties without the constraints of arbitrary windowing assumptions.

**Objective:**

To characterize dynamic functional connectivity alterations in young patients with migraine without aura (MWoA) using HMM and examine associations between dynamic state metrics and clinical disability.

**Methods:**

Resting-state fMRI data were obtained from 200 participants (100 young MWoA patients and 100 matched healthy controls). Using the Dosenbach 160 ROI template (cerebellum excluded; *N* = 142), HMM identified recurring brain states. Group differences in fractional occupancy (FO), mean dwell time (MDT), and state transition probabilities were assessed. State-specific functional connectivity patterns were compared, and correlations with clinical indices (MIDAS, VAS, HIT-6) were evaluated.

**Results:**

Eleven robust dynamic brain states were identified. Compared with controls, migraine patients demonstrated increased FO and MDT in States 7 (dorsal attention network–dominant) and 9 (multisensory integration), alongside reduced values in sensorimotor states (States 3, 4, 8, 11). State 9 exhibited significant abnormalities in DMN–SC and DMN–VAN connectivity (FDR-corrected *q* < 0.05). Transition analyses revealed enhanced self-transitions and increased incoming transitions to States 7 and 9, whereas transitions among sensorimotor states were diminished. MDT in State 9 was positively correlated with MIDAS scores (*r* = 0.38, *p* < 0.05), indicating its association with functional disability.

**Conclusions:**

Young MWoA patients exhibit a dual-mode dysfunction in brain dynamics: heightened external vigilance (State 7) and impaired segregation of internal–external processing (State 9), accompanied by instability in baseline sensorimotor configurations. Prolonged dwelling in State 9 and its correlation with disability highlight this multisensory integration state as a potential biomarker of migraine-related functional impairment. These findings provide new insights into neurobiological mechanisms and support dynamic network–based therapeutic strategies.

## 1 Introduction

Migraine is a prevalent primary neurological disorder associated with high disability, particularly among young and middle-aged individuals (Amiri et al., 2021). According to the Global Burden of Disease Study 2019, migraine ranks as the second leading cause of years lived with disability (YLDs) worldwide (Steiner et al., 2020). Migraine affects ~10–12% of the general population worldwide, with the highest prevalence observed in adolescents and young adults, among its subtypes, migraine without aura (MwoA) is the most common, accounting for ~70–75% of all migraine cases (Cloet et al., 2024; Al-Hashel et al., 2022; Zhao Q. et al., 2021). Its recurrent and long-term attacks substantially impair patients' academic performance, work productivity, and overall quality of life. Despite ongoing advances in clinical diagnosis and therapeutic strategies, the neurobiological mechanisms underlying migraine remain incompletely understood (Schwedt et al., 2015). In recent years, neuroimaging research has offered new insights into the central mechanisms of migraine, with functional magnetic resonance imaging (fMRI) becoming a widely used tool for characterizing abnormal patterns of brain network connectivity (Skorobogatykh et al., 2019).

Traditional resting-state fMRI studies primarily rely on static functional connectivity (sFC) analysis, which calculates the average correlation between brain regions across the entire scanning period (Zacher et al., 2017). Using this approach, researchers have identified widespread connectivity abnormalities in migraine patients involving multiple large-scale networks, including the default mode network (DMN), salience network (SN), and sensorimotor network (SMN; Hu et al., 2023). Notably, studies focusing specifically on MwoA have further demonstrated disrupted connectivity within pain-processing, affective, and attentional circuits, including altered thalamo-cortical interactions, decreased coherence within the DMN, and aberrant coupling between the SN and insular cortex, suggesting subtype-related functional impairments (Gao et al., 2016). However, mounting evidence indicates that functional connectivity is inherently dynamic, fluctuating and reorganizing over time rather than remaining static (Zou et al., 2021). Static analyses fail to capture these temporal variations and may obscure transient neural activity patterns relevant to disease mechanisms (Zou et al., 2021). Therefore, investigating the dynamic functional connectivity (dFC) of brain networks is essential for achieving a more comprehensive understanding of the neurobiological underpinnings of migraine (Zhou et al., 2023).

Analytical approaches targeting dynamic brain features have progressed rapidly, with the sliding-window method being one of the earliest and most widely used techniques (Ingram et al., 2024). By applying a fixed temporal window to the fMRI time series, this method generates connectivity matrices for different time segments, thereby describing temporal variability in brain networks (Savva et al., 2019). Nonetheless, the sliding-window approach has notable limitations, including subjective window-length selection and restricted temporal resolution, which hinder its ability to detect rapid neural state transitions (Vergara et al., 2019). In contrast, the Hidden Markov Model (HMM) provides a more advanced probabilistic framework that can infer latent “brain states” directly from time series data without predefined windowing assumptions (Hussain et al., 2023). Each inferred state represents a stable and characteristic functional pattern, while the temporal occurrence, duration, and transitions between states reflect intrinsic dynamic properties of brain networks. Recent studies have successfully applied HMM to fMRI data in healthy individuals and in patients with Alzheimer's disease, depression, and epilepsy, demonstrating its superior sensitivity and interpretability (Geng et al., 2025).

In the context of migraine, previous studies have reported significant abnormalities in dynamic brain activity. For instance, sliding-window analyses have revealed increased temporal variability in pain-related and attention networks, suggesting reduced network flexibility in migraine patients (Lee et al., 2019). In MwoA, dynamic analyses have revealed abnormal functional activity within the periaqueductal gray, limbic regions, and the default mode network, suggesting instability in pain modulation and maladaptive transitions between internally and externally oriented cognitive state (Tessitore et al., 2013). To the best of our knowledge, HMM has not yet been widely applied to systematically characterize whole-brain resting-state fMRI–derived dynamic connectivity states in migraine; existing dFC studies have primarily relied on sliding-window or related approaches (Lee et al., 2019). This gap is particularly relevant in young individuals, whose heightened neural plasticity may lead to more pronounced early functional alterations that better reflect disease-related changes. Given that migraine is characterized by episodic, fluctuating, and network-level dysregulation, HMM-based analysis may reveal latent dynamic brain state patterns and elucidate abnormal state transitions in affected individuals (Jiang et al., 2022).

Accordingly, the present study applies the Hidden Markov Model to resting-state fMRI data from young migraine patients. Our aims are threefold: (1) to identify characteristic dynamic brain states in young migraine patients and healthy controls; (2) to compare group differences in state fractional occupancy (FO), mean dwell time (MDT), and switching rate; and (3) to further explore associations between these dynamic indices and clinical measures such as headache severity, attack frequency, and functional impact. We hypothesize that young migraine patients will exhibit abnormal state distributions and transition patterns, particularly in states involving the salience network and default mode network. Such dynamic imbalances may reflect impairments in pain processing, emotional regulation, and internal–external attention switching, offering new neuroimaging evidence for understanding the central mechanisms of migraine and informing early intervention strategies.

## 2 Methods

### 2.1 Subject recruitment and grouping

Participants were consecutively recruited from January 1, 2020 to December 14, 2022 at Shuguang Hospital affiliated with Shanghai University of Traditional Chinese Medicine (Shanghai, China). All patients met the diagnostic criteria for MwoA according to the third edition of the International Classification of Headache Disorders (ICHD-3) and underwent MRI scanning during the interictal phase (Olesen, 2018). The two groups were matched in age, gender, and years of education. Healthy controls (HCs) were screened to ensure no history of migraine or other recurrent primary headache disorders, and no first-degree family history of migraine. HCs were also excluded if they met any of the general exclusion criteria listed below (neurological/psychiatric disorders, major systemic diseases, structural brain abnormalities on imaging, MRI contraindications, or use of medications affecting the central nervous system). The inclusion criteria were as follows: (1) age between 18 and 35 years; (2) right-handedness; (3) a minimum of 9 years of education; (4) fulfillment of the ICHD-3 diagnostic criteria for “Migraine without Aura (1.1)” in the MwoA group, with a disease duration ≥ 1 year and a monthly attack frequency of 1–8 episodes; (5) absence of headache attacks within the 72 h preceding the scan and no use of analgesics or medications affecting the central nervous system within the past 7 days and being headache-free at the time of scanning; (6) ability to understand and comply with all study procedures.

Exclusion criteria were: comorbid neurological or psychiatric disorders (e.g., epilepsy, depression/anxiety) or major systemic diseases; history of traumatic brain injury, cerebrovascular disorders, intracranial tumors, or any imaging-detected structural abnormalities; contraindications to MRI (e.g., metal implants, severe claustrophobia); and overuse of headache medications or recent preventive treatment for headaches within 3 months prior to MRI scanning. All participants provided written informed consent. The study protocol was approved by the Ethics Committee of Shuguang Hospital affiliated to Shanghai University of Traditional Chinese Medicine Clinical Trial Registry Platform (World Health Organization under registration number: 2019-766-121-01) and conducted in accordance with the Declaration of Helsinki.

### 2.2 Clinical assessment protocols

Baseline clinical evaluations were performed for all participants using the following standardized scales:

SDS (Self-Rating Depression Scale): A 20-item self-report questionnaire evaluating depressive symptoms over the previous week, with each item rated from 1 to 4. Standardized scores were calculated according to the scale manual, with scores ≥ 50 indicating depressive tendencies (Zung, 1965).

VAS (Visual Analog Scale): A 0–10 scale used to assess the intensity of pain experienced during typical migraine attacks, where higher scores reflect more severe pain (Huskisson, 1974).

HIT-6 (Headache Impact Test-6): A six-item instrument assessing the impact of headaches on daily life and overall functioning, with total scores ranging from 36 to 78. Higher scores indicate greater functional impairment (Kosinski et al., 2003).

MIDAS (Migraine Disability Assessment Scale): A five-item scale quantifying the number of days within the past 3 months during which headaches caused functional impairment. Scores of 0–5 indicate mild disability, 6–10 moderate disability, and ≥ 11 severe disability (Stewart et al., 2001). The total MIDAS score was categorized into four grades: 0–5 (Grade I, little or no disability), 6–10 (Grade II, mild disability), 11–20 (Grade III, moderate disability), and ≥21 (Grade IV, severe disability).

All clinical assessments (SDS, VAS, HIT-6, and MIDAS) were administered on the same *day* as MRI and completed prior to scanning during the interictal period (confirmed according to the study criteria). Questionnaires were completed using *paper*-based, self-administered forms in a quiet room. A trained researcher provided standardized instructions and checked the questionnaires for completeness immediately after completion without coaching responses. The recall periods were as follows*: S*DS assessed depressive symptoms over the past *week*; VAS captured the pain intensity of a typical migraine attack (0 = no pain*; 1*0 = worst imaginable pain); HIT-6 evaluated headache-related impact over the past 4 *weeks*; and MIDAS quantified migraine-related disability over the past 3 *months*.

### 2.3 MRI data acquisition and resting-state instructions

MRI data were acquired on a 3.0-T scanner (uMR 790, Shanghai United Imaging Healthcare) using a 32-channel head coil. High-resolution T1-weighted structural images were obtained using a T1-weighted structural sequence with the following parameters: TR = 7.2 ms, TE = 3.1 ms, flip angle = 10°, FOV = 256 × 256 mm^2^, matrix = 512 × 512, slice thickness = 1 mm, and 192 slices. Resting-state fMRI data were collected using a gradient-echo echo-planar imaging (EPI) sequence: TR = 2,000 ms, TE = 30 ms, flip angle = 90°, FOV = 224 × 224 mm^2^, matrix = 64 × 64, slice thickness = 3.5 mm, 33 slices, voxel size = 3.5 × 3.5 × 3.5 mm3, and 240 volumes (total scan duration = 8 min). During the resting-state scan, participants were instructed to relax, remain as still as possible, and stay awake throughout the entire acquisition.

### 2.4 MRI data preprocessing

**Figure 1:**
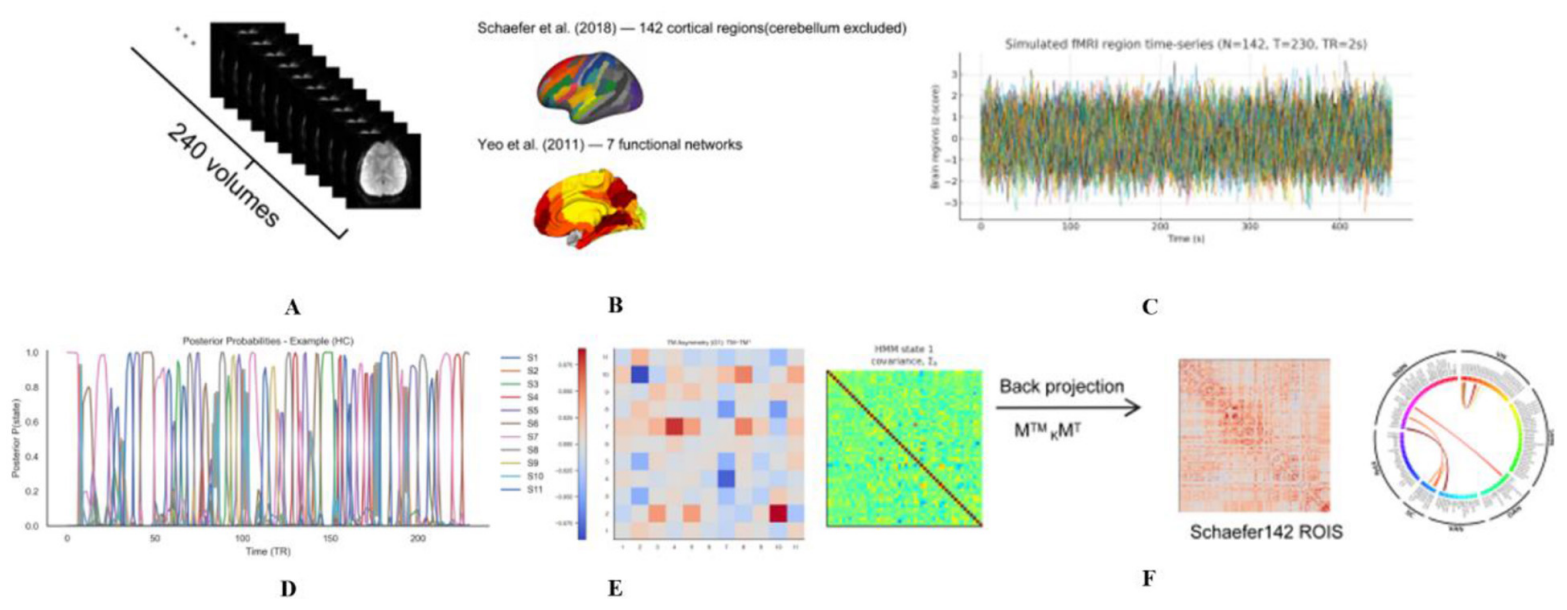
Schematic workflow of HMM-based functional dynamic analysis. **(A–C)** Data Preprocessing: resting-state fMRI data (240 volumes) were parcellated into 142 ROIs (Schaefer atlas) within 7 functional networks (Yeo atlas). Standardized BOLD time-series were then extracted. **(D)** State Inference: the HMM was applied to identify 11 latent brain states. The plot displays the time-varying posterior probabilities of states for a representative subject. **(E)** Transition Matrix: the heatmap illustrates the group-level transition probabilities between states, where each cell represents the likelihood of switching from one state to another. **(F)** Spatial Patterns: state-specific covariance matrices were estimated and visualized as heatmaps and chord diagrams to characterize the functional connectivity architecture of each state. fMRI, functional magnetic resonance imaging; HMM, hidden Markov model; ROI, region of interest; S1–S11 States 1 to 11.

Preprocessing was performed using DPABI_V9.0_250415 and included the following steps: (1) removal of the first ten time points to eliminate magnetization equilibration effects; (2) slice timing and head-motion correction; (3) co-registration of functional images to each participant's T1-weighted image, followed by normalization to MNI space; (4) spatial smoothing with a Gaussian kernel (FWHM = 6 mm); (5) linear detrending and band-pass filtering (0.01–0.08 Hz); (6) regression of confounding variables, including Friston-24 head-motion parameters, white matter, and cerebrospinal fluid signals, with no global signal regression; and (7) quality control, whereby participants were excluded or data were scrubbed if mean framewise displacement (FD) exceeded 0.2 mm or more than 20% of volumes exceeded FD > 0.5 mm. FD was calculated to quantify in-scanner head motion and was used for quality control and motion characterization across participants. An overview of the study workflow is shown in Figure 1.

### 2.5 ROI definition and time series extraction

Whole-brain parcellation was performed using the DPABI-distributed Dosenbach 160 ROI template (cerebellum excluded; N = 142; Dosenbach et al., 2010). To avoid potential cerebellar confounds and to focus on cerebral networks, we used the cerebellum-excluded version of this template, resulting in 142 ROIs (*N* = 142) spanning cortical and subcortical regions. Preprocessed mean time series were extracted for each ROI. Preprocessed time series for each ROI were extracted. For each participant, pairwise Pearson correlation coefficients were computed between all ROI time series to construct a 142 × 142 ROI-by-ROI functional connectivity (FC) matrix (symmetric correlation matrix). Correlation coefficients were then Fisher z-transformed, and diagonal elements were set to zero.

### 2.6 Hidden Markov model (HMM)

#### 2.6.1 Input and standardization

For each participant, ROI time series were z-standardized. Time series from all participants were then concatenated into a single sequence, and the length of each individual's continuous data segment (after scrubbing) was recorded to ensure that transitions were constrained to occur only within segments from the same participant.

#### 2.6.2 State determination

A grid search was conducted across *K* = 2–14 states, selecting the optimal number primarily based on the global minimum Bayesian Information Criterion (BIC), together with the log-likelihood profile to assess diminishing returns as K increased. The final model used K = 11 states (Supplementary Figure 1). The model was repeated ten times with different random seeds, and the solution with the highest log-likelihood was used for downstream analyses. Because HMM state labels are permutation-invariant, states were aligned across runs using maximum-correlation matching (Hungarian assignment) based on vectorized state-wise FC patterns, and stability results are reported in Supplementary Figure 2.

#### 2.6.3 Model setup and fitting

A Gaussian HMM (covariance_type = “full”) was fitted to the concatenated sequence using the Expectation–Maximization (EM) algorithm, with a maximum of 500 iterations and a fixed random seed. The lengths vector was included to account for time discontinuities due to scrubbing and to prevent spurious transitions between participants.

#### 2.6.4 State inference and transition matrix

The most probable state sequence (Viterbi path) and posterior probabilities γ(t, k) were computed for each time point. Transition frequencies for each directional transition (i → j) were derived and normalized to obtain individual transition probability matrices (TPMs). A group-average TPM was generated by averaging across participants for visualization and group-level comparisons.

#### 2.6.5 Individualized metrics and robustness assessment

Individual metrics—including fractional occupancy (FO), mean dwell time (MDT), transition counts, and individualized TPMs—were computed using γ(t, k) and the Viterbi sequence. To evaluate robustness, PCA was used to reduce the dimensionality to 30 components, and models were re-estimated within a *K* ± 2 range, yielding consistent state patterns.

#### 2.6.6 State-specific individual connectivity matrices

For each participant and each state, ROI × ROI connectivity matrices were calculated from time points assigned to that state, using a minimum frame threshold of 30. Covariance estimation was performed using Ledoit–Wolf shrinkage, followed by conversion to correlation matrices and Fisher z-transformation. If a participant did not meet the minimum frame requirement for a given state, the matrix was marked as missing, and only available data were included in state-wise statistical analyses.

#### 2.6.7 Definition of high/neutral/low (relative network load)

For each HMM state, we first summarized the connectivity strength at the level of seven large-scale networks by averaging connectivity values within each network (based on the ROI-to-network assignment). We then computed a state-specific baseline by taking the mean of the seven network-average values within the same state. The relative load of each network in that state was defined as its deviation from this state-specific baseline: networks above the baseline were labeled High, and networks below the baseline were labeled Low. To improve interpretability and avoid the influence of extreme values, the relative deviations were capped within ±50% (i.e., values higher than +50% were set to +50%, and values lower than −50% were set to −50%). Finally, the overall High/Neutral/Low pattern of each state was summarized by counting how many networks were labeled High vs. Low (Neutral networks were those close to the baseline and did not show an obvious elevation or reduction), which was then used for the descriptive categorization of the 11 states.

### 2.7 Statistical analysis

Primary group comparisons were conducted without additional covariate adjustment, as the two groups were matched on key demographics (age/sex/education). We acknowledge that residual confounding effects of head motion and mood cannot be fully excluded. Continuous variables (e.g., age, disease duration, monthly headache days, VAS, HIT-6, MIDAS, SDS scores) were compared between groups using one-way ANOVA. Categorical variables (e.g., gender) were compared using Chi-square tests. *Post hoc* pairwise comparisons, when required, were adjusted using Bonferroni correction. Statistical significance was set at *p* < 0.05, and results were reported as mean ± standard deviation or median.

#### 2.7.1 Within-state individual connectivity analysis

For each state k, edge-wise connectivity strengths were compared between the MwoA and HC groups using two-sided Welch's *t*-tests to account for heteroscedasticity. Only participants with ≥ 30 valid frames in a given state were included. Raw *p*-values across all “state × edge” tests were corrected for false discovery rate (FDR) using the Benjamini–Hochberg method, with significance defined as *q* < 0.05.

#### 2.7.2 Group comparisons of FO, MDT, and switching rate

Independent-samples *t*-tests were used for normally distributed, homoscedastic variables, whereas Mann–Whitney U tests were applied for non-normal data. Statistical significance was set a nominal threshold of two-tailed *p* (uncorrected) < 0.05, with results summarized as mean ± standard deviation or median. Because FO, MDT, and switching rate were examined across multiple HMM states, no multiple-comparison correction was applied for these state-wise tests; therefore, the findings should be considered exploratory/hypothesis-generating.

#### 2.7.3 Transition probability comparisons

Transition probabilities for directed transitions Si → S j were analyzed after applying arcsine square-root transformations to stabilize variance, followed by independent-samples *t*-tests. Mann–Whitney U tests were employed when data deviated from normality assumptions. To account for multiple comparisons across all edges, the Benjamini–Hochberg procedure was used to control the False Discovery Rate (FDR). Statistical significance was defined as an FDR-corrected *p*-value (represented as *q*) < 0.05.

#### 2.7.4 Clinical correlation

At the individual level, correlations were assessed between FO, MDT, transition counts, and clinical variables (VAS, HIT-6, MIDAS, SDS, disease duration, monthly headache days). Pearson's correlation was used for normally distributed variables, and Spearman's correlation for non-normally distributed variables. Clinical correlation analyses were performed as exploratory analyses without covariate adjustment. SDS scores were collected to characterize depressive symptoms, but were not included as covariates in the correlation models; therefore, potential mood-related confounding of disability measures (e.g., MIDAS/HIT-6) cannot be fully excluded. For clinical correlation analyses, results were reported at a significance level of *p* < 0.05 (uncorrected). Given the exploratory nature of these analyses, we reported these nominal associations to guide future hypothesis-driven research.

## 3 Results

### 3.1 Subject characteristics

A total of 200 eligible participants were included in this study, comprising 100 young MwoA patients (MwoAs) and 100 HCs. The two groups were well matched with respect to demographic variables. Detailed demographic and clinical characteristics are presented in Table 1.

**Table 1:** Demographic information and clinical scale assessments of the study participants.

**Variable**	**HCs (*n* = 100)**	**MwoAs (*n* = 100)**	**Statistic**	***p*-value**
Age (years)	28.1 ± 6.2	28.1 ± 5.8	*t* = −0.01	0.99
Sex (M/F)	48/52	46/54	χ^2^ = 0.02	0.89
Education (years)	15.8 ± 4.3	16.0 ± 4.4	*t* = −0.36	0.72
Disease duration (years)	—	6.6 ± 4.9	—	—
SDS (score)	—	49.8 ± 6.3	—	—
VAS (score)	—	5.6 ± 1.8	—	—
HIT-6 (score)	—	52.3 ± 9.0	—	—
MIDAS (score)	—	18.8 ± 9.2	—	—

M, male; F, female; SDS, self-rating depression scale; VAS, visual analog scale; HIT-6, headache impact test-6; MIDAS, migraine disability assessment. t, student's *t*-test; χ^2^, chi-square test. “—” indicates not applicable.

### 3.2 HMM state characteristics and spatial distribution

**Figure 2:**
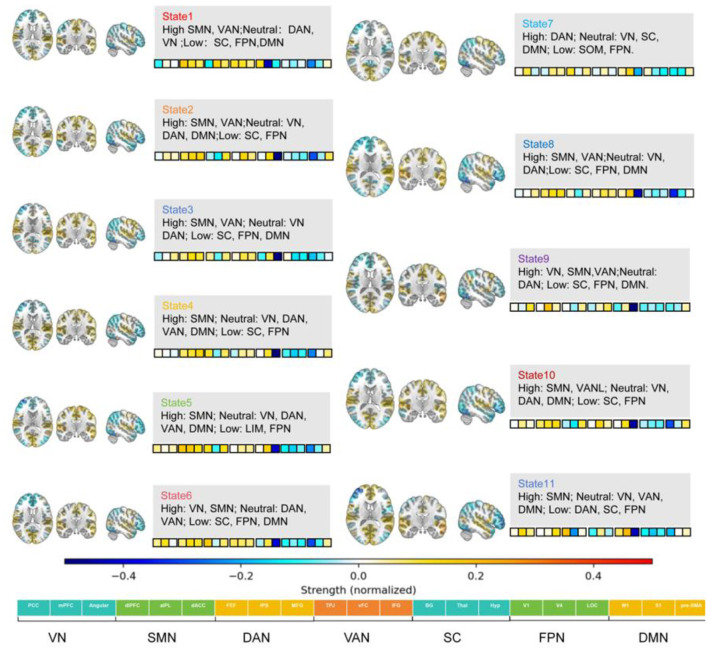
Spatial characterization and network architecture of the 11 inferred HMM states. State Profiles: For each state (state 1–state 11), the spatial maps **(left)** illustrate the mean activation/connectivity strength back-projected onto brain surfaces. Network-Level Features: the accompanying text boxes categorize functional networks into “High,” “Neutral,” or “Low” based on their relative contribution to each state. ROI-to-Network Mapping: the color-coded grid at the bottom defines the correspondence between individual ROIs and the seven canonical functional networks. Color Scale: the color bar (ranging from −0.4 to 0.4) represents the normalized connectivity strength, where warm colors (red/yellow) indicate increased and cool colors (blue) indicate decreased functional load. VN, visual network; SMN, somatomotor network; DAN, dorsal attention network; VAN, ventral attention network; SC, subcortical; FPN, frontoparietal network; DMN, default mode network; ROI, region of interest.

In the grid search across *K* = 2–14, the Bayesian Information Criterion (BIC)reached its minimum at *K* = 11. Considering the marginal improvement in log-likelihood and the biological interpretability of resulting patterns, the final model retained 11 hidden states (State 1–11; see Figure 2). Each state exhibited a stable and clearly distinguishable spatial configuration on the whole-brain 142 × 142 ROI functional connectivity matrices, indicating the presence of multiple reproducible network organization patterns during resting state.

Based on the relative load (High/Neutral/Low) across large-scale networks, the 11 states were classified into four categories.

#### 3.2.1 SMN + VAN dominant type (States 1, 2, 3, 8, 10)

These states collectively showed elevated loadings in the Sensorimotor Network (SMN) and the Salience/Ventral Attention Network (VAN). The Visual Network (VIS) and/or Dorsal Attention Network (DAN) remained predominantly neutral, whereas the Subcortical System (SC), Frontoparietal Network (FPN), and Default Mode Network (DMN) exhibited lower loadings.

#### 3.2.2 SMN single-dominant type (States 4, 5, 11)

These states were characterized by pronounced increases in SMN/SOM activity, with VIS, DAN, VAN/SAL, and DMN largely neutral. The Limbic Network (LIM) and FPN showed lower loadings. Notably, State 11 also demonstrated suppressed DAN activity.

#### 3.2.3 VN–SMN coordinated type (States 6, 9)

State 6: Elevated activity in both VN and SMN, while DAN and VAN were neutral, and SC, FPN, and DMN were lower.

State 9: Concurrent elevation in VN, SMN, and VAN, with neutral DAN and reduced SC, FPN, and DMN activity.

#### 3.2.4 DAN dominant type (state 7)

This state exhibited increased loading in DAN, with neutral activity in VIS, VAN, SC, and DMN, and decreased activity in SMN and FPN.

**Abbreviations:** DMN = Default Mode Network; FPN = Frontoparietal Network; DAN = Dorsal Attention Network; VAN = Ventral Attention Network; VN = Visual Network; SMN = Sensorimotor Network; SC = Subcortical System.

### 3.3 Comparison of within-state individual connectivity between groups

**Figure 3:**
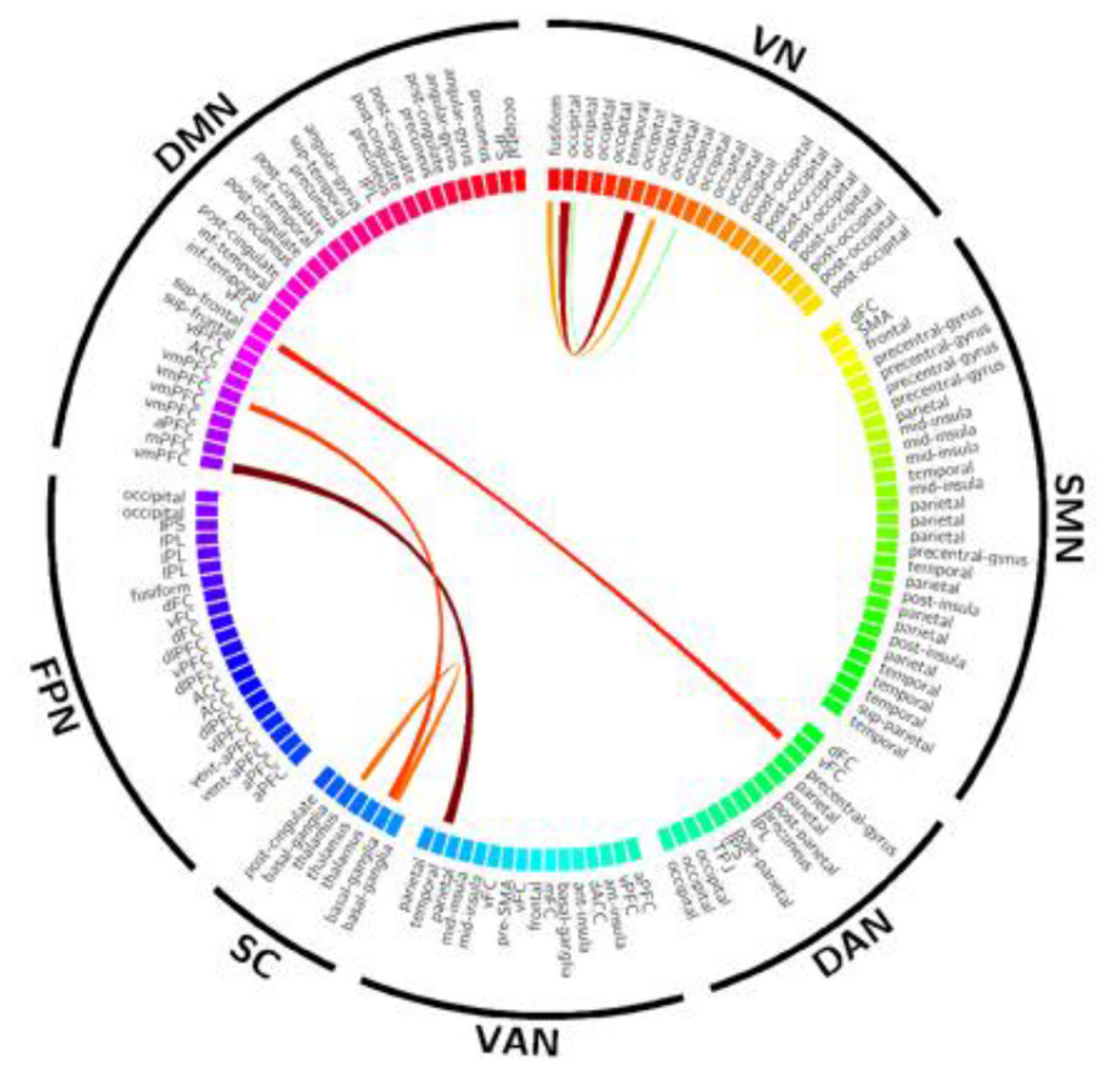
Group differences in functional connectivity within State 9. Chord Diagram: illustrates significant edge-to-edge functional connectivity (FC) alterations in migraine patients relative to healthy controls (HCs) during State 9. Nodes and Networks: 142 ROIs are organized into 7 functional networks as color-coded on the circular perimeter. Statistical threshold: only connections surviving Benjamini-Hochberg FDR correction (*q* < 0.05) are displayed. Color Coding: red arcs denote increased FC in patients (Patients >HCs) while blue arcs (if present) denote decreased FC (Patients < HCs) Arc thickness represents the magnitude of the t-statistic. VN, visual network; SMN, somatomotor network; DAN, dorsal attention network; VAN, ventral attention network; SC, subcortical network; FPN, frontoparietal network; DMN, default mode network; ROI, region of interest.

Significant group differences were detected in **State 9** at the edge level (Welch's *t*-test with Benjamini–Hochberg FDR correction, *q* < 0.05). These differences were primarily localized within cross-network connections involving the DMN–SC, and extended to DMN–VAN as well as Visual Network (VN) connectivity (Figure 3). These findings indicate disrupted coupling patterns in this state, particularly affecting sensorimotor–default network regulatory dynamics.

### 3.4 Temporal dynamics of HMM states

**Figure 4:**
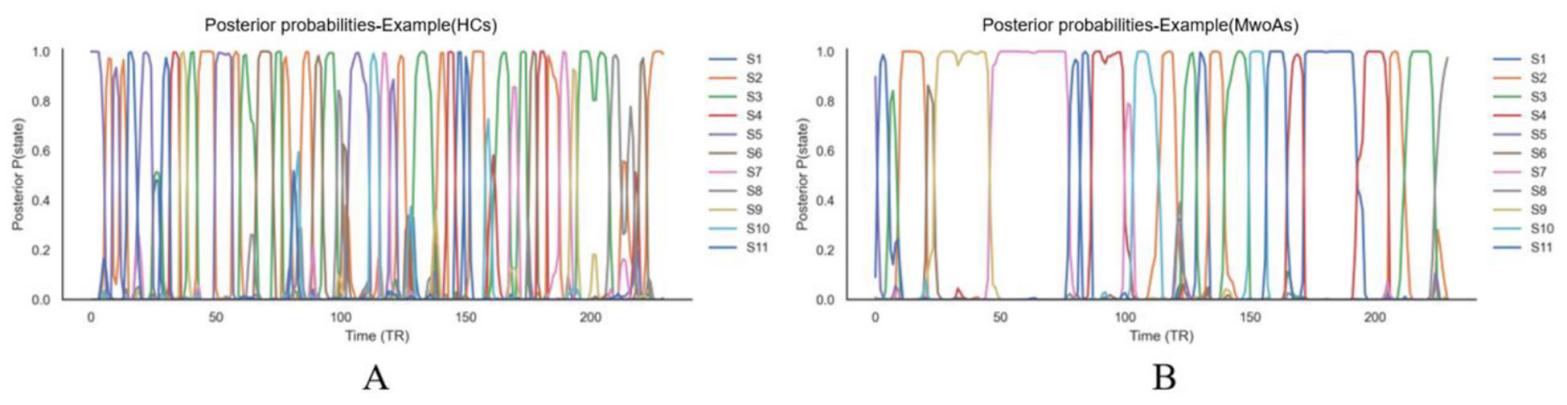
Representative temporal dynamics of HMM state probabilities. State Time-courses: the plots illustrate the posterior probability sequences of 11 inferred HMM states (S1–S11) for an exemplary **(A)** Healthy Control (HC) and **(B)** Patient with Migraine without Aura Axes Definition: the vertical axis (y-axis) indicates the posterior probability of each state ranging from 0 to 1; the horizontal axis (x-axis) represents time in Repetition Times (TRs). State Switching: rapid fluctuations between peaks of different colored lines denote the dynamic switching of the brain's latent functional states over the scan duration. HMM, hidden Markov model; HC, healthy control; MwoA, migraine without aura; TR, repetition time S1–S11 States 1 to 11.

The HMM generated posterior state-probability sequences (γ) for each participant, reflecting the moment-to-moment probability of occupying each state throughout the scanning period (Figure 4A HCs; Figure 4B MwoAs).

### 3.5 FO and MDT

#### 3.5.1 FO

**Figure 5:**
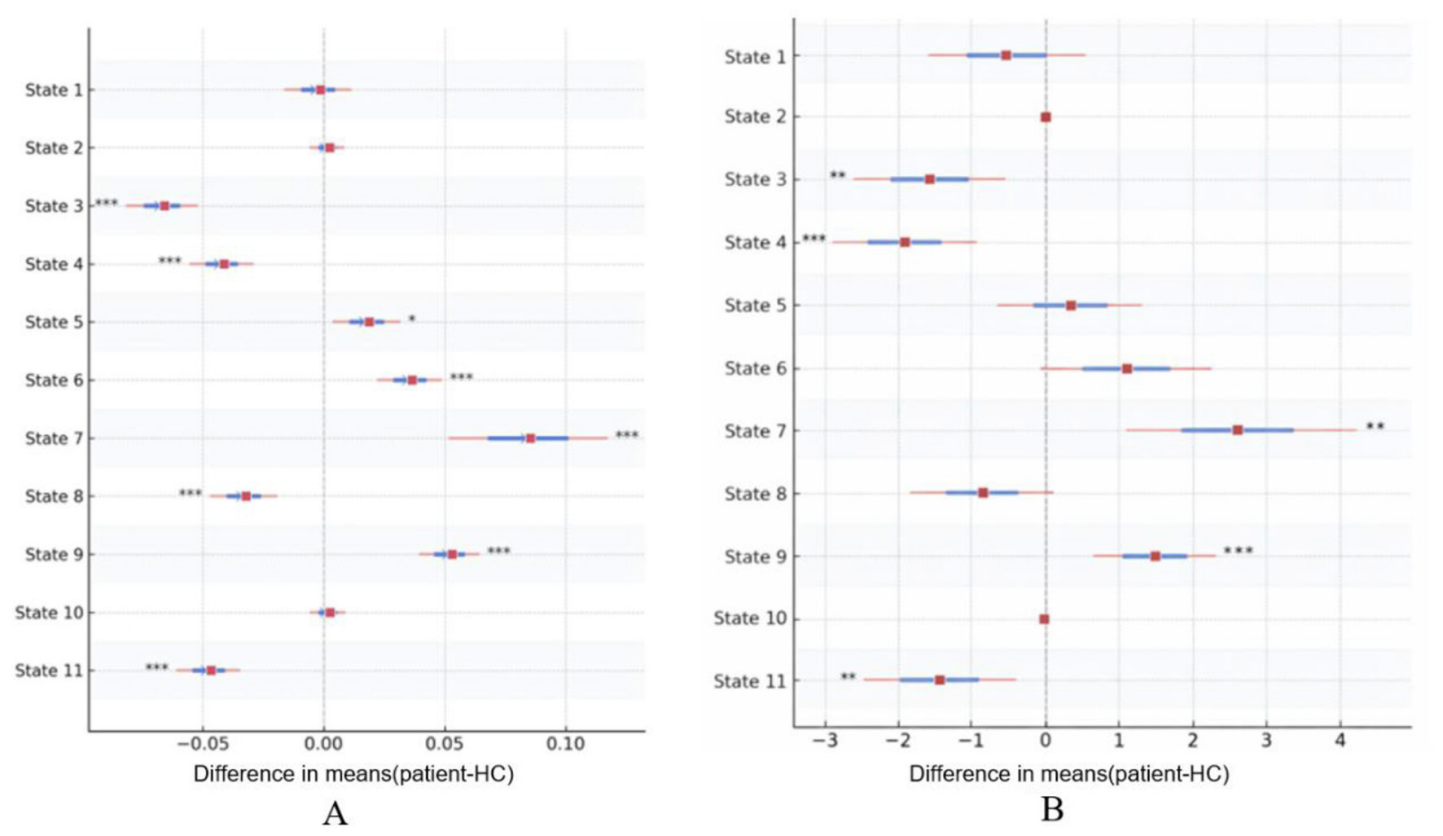
Groups Differences in FO and MDT. Comparison: forest plots show mean group differences (patients minus HCs) for **(A)** fractional occupancy (FO) and **(B)** mean dwelling time (MDT). Symbols: squares represent point estimates; horizontal lines indicate 95% confidence intervals (CIs). Values > 0 denote higher metrics in patients. Statistics: asterisks indicate significant differences after multiple comparison correction: *p < 0.05, **p < 0.01, ***p < 0.001. FO, fractional occupancy; MDT, mean dwelling time; TR, repetition time; HCs, healthy controls.

Compared with healthy controls, the young migraine group exhibited increased FO in States 6, 7, 9, and 5, whereas FO was decreased in States 3, 4, 8, and 11. No significant group differences were observed for States 1, 2, and 10 (Figure 5A, Table 2).

**Table 2:** Fractional occupancy (FO) by state (HC vs. patients).

**State**	**HC (mean ±SD)**	**Patients (mean ±SD)**	**Stat**	***P***	**q (FDR)**	**Effect**	**Cohen's *d***
S1	0.089 ± 0.049	0.087 ± 0.050	5,100	0.8078	0.8078	−0.0200	−0.05
S2	0.056 ± 0.025	0.057 ± 0.027	4,741.5	0.5278	0.5904	0.0517	0.05
S3	0.138 ± 0.061	0.071 ± 0.044	8,154.5	1.26e-14	1.39e-13	−0.6309	−1.26
S4	0.127 ± 0.048	0.085 ± 0.047	7,325.5	1.32e-08	2.90e-08	−0.4651	−0.89
S5	0.100 ± 0.048	0.118 ± 0.053	3,987.5	0.0134	0.0184	0.2025	0.35
S6	0.075 ± 0.041	0.111 ± 0.054	2,984	8.35e-07	1.53e-06	0.4032	0.74
S7	0.051 ± 0.049	0.136 ± 0.159	2,322	5.93e-11	2.17e-10	0.5356	0.72
S8	0.130 ± 0.050	0.097 ± 0.049	6,770	1.52e-05	2.39e-05	−0.3540	−0.67
S9	0.058 ± 0.039	0.110 ± 0.050	2,051.5	5.74e-13	3.15e-12	0.5897	1.16
S10	0.057 ± 0.025	0.058 ± 0.028	4,747	0.5367	0.5904	0.0506	0.06
S11	0.117 ± 0.050	0.070 ± 0.044	7,616	1.61e-10	4.43e-10	−0.5232	−1.01

Cohen's *d* is computed as an equal-*n* approximation: *d* ≈ (Patients – HC)/sqrt ((SD_HC^2^ + SD_Pt^2^)/2).

#### 3.5.2 MDT

Relative to healthy controls, MDT was significantly increased in States 7 and 9, but was reduced in States 3, 4, 11, and 10. The remaining states (States 1, 2, 5, 6, and 8) demonstrated no significant between-group differences (Figure 5B, Table 3).

**Table 3:** Mean dwell time (MDT) by state (HC vs. patients).

**State**	**HC (mean ±SD)**	**Patients (mean ±SD)**	**Stat**	***P***	**q (FDR)**	**Effect**	**Cohen's *d***
S1	6.87 ± 4.14	6.34 ± 3.14	5,156.5	0.7030	0.7030	−0.0313	−0.14
S2	1.00 ± 0.00	0.97 ± 0.17	5,150	0.0827	0.1138	−0.0300	−0.25
S3	8.05 ± 3.72	6.46 ± 3.52	6,340	0.0011	0.0023	−0.2680	−0.44
S4	8.38 ± 3.83	6.46 ± 3.01	6,428.5	0.0005	0.0018	−0.2857	−0.56
S5	7.05 ± 3.45	7.35 ± 3.58	4,799	0.6240	0.6865	0.0402	0.09
S6	6.25 ± 3.24	7.32 ± 4.86	4,347	0.1107	0.1353	0.1306	0.26
S7	3.84 ± 1.98	6.38 ± 7.27	3,126	4.63e-06	5.09e-05	0.3748	0.48
S8	7.76 ± 3.39	6.88 ± 3.54	5,809.5	0.0480	0.0755	−0.1619	−0.25
S9	5.58 ± 3.17	7.02 ± 2.83	3,455	0.0002	0.0009	0.3090	0.48
S10	1.00 ± 0.00	0.96 ± 0.20	5,200	0.0444	0.0755	−0.0400	−0.29
S11	7.77 ± 3.45	6.34 ± 3.96	6,349.5	0.0010	0.0023	−0.2699	−0.38

Cohen's *d* is computed as an equal-n approximation: *d* ≈ (Patients – HC)/sqrt((SD_HC^2^ + SD_Pt^2^)/2).

### 3.6 State transition probability matrix

**Figure 6:**
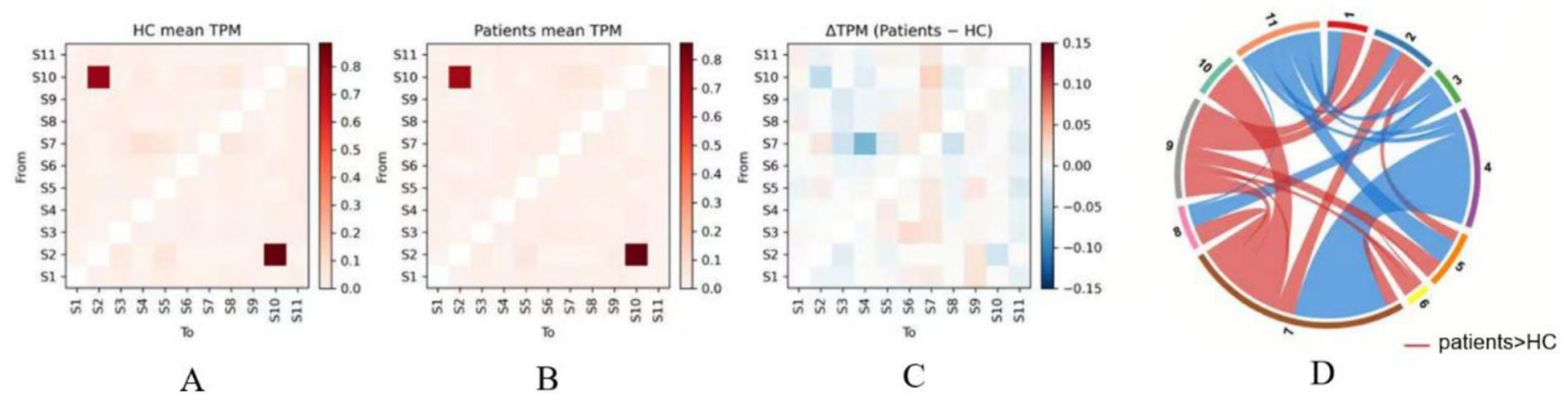
Group comparisons of HMM state transition dynamics. **(A, B)** group mean TPM: transition probability matrices for healthy controls (HCs) and migraine patients. Each cell (i, j) represents the probability of transitioning from State i (row) to State j (column). The color bar (0.0–0.8) indicates the probability magnitude. **(C)** Difference matrix: illustrates the net difference in transition probabilities (patients minus HCs). Red and blue cells denote increased and decreased transition likelihoods in the patient group, respectively. **(D)** Significant transitions: chord diagram highlighting state transition pathways that differ significantly between groups (FDR-corrected, *q* < 0.05). Color and line weight: red arcs indicate higher transition rates in patients (Patients > HCs), while blue arcs indicate higher rates in controls (HCs > Patients). Line thickness is proportional to the effect size of the group difference. TPM, transition probability matrix; HMM, hidden Markov model; HC, healthy control; FDR, false discovery rate; S1–S11, States 1 to 11.

Figures 6A–C presents the mean transition probability matrices for healthy controls and headache patients, along with their differences. Both groups exhibited similar dominant transition patterns, with S10 → S2 and S2 → S10 showing the highest transition probabilities.

Figure 6D between-group difference analysis (ΔTPM = Patients - HC) with FDR correction revealed significant state transition differences (*q* < 0.05). In healthy controls, both in-degree and out-degree of S11 were significantly higher than in patients, the transition S7 → S4 showed the largest difference, and transitions such as S8 → S3 were also significantly stronger in healthy controls. In contrast, in headache patients, both in-degree and out-degree of S7 and S9 were significantly enhanced. Multiple transition pathways directing toward S7 and S9 were significantly increased in patients, including S10 → S7, S8 → S7, S9 → S7, and S1 → S9, S2 → S9, S5 → S9.

### 3.7 Clinical correlation

**Figure 7:**
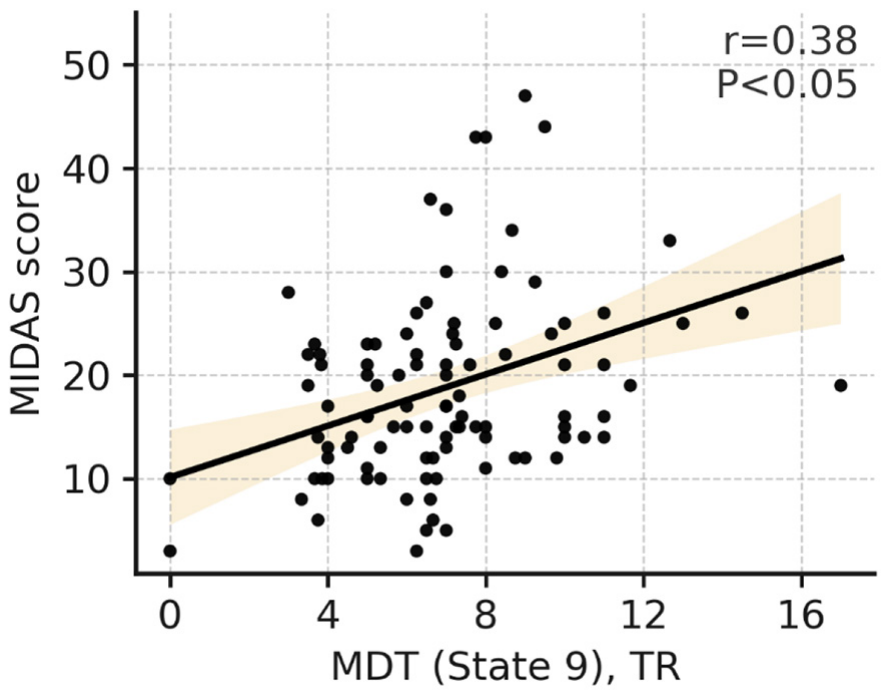
Correlation between MDT (State 9) and MIDAS score. Scatter plot: each point represents one patient. The x-axis is the MDT of State 9 (in TRs); the y-axis is the total MIDAS score. Regression: the solid line represents the least-squares fit; the shaded area represents the 95% confidence interval. Statistics: pearson correlation coefficient and two-tailed *p-*value are provided. MIDAS, Migraine Disability Assessment; MDT, mean dwelling time; TR, repetition time.

At the individual level, MDT in **State 9** was positively correlated with MIDAS scores (*r* = 0.38, *p* < 0.05, uncorrected). No significant correlations were observed between the remaining FO or MDT measures and clinical indices, including VAS, HIT-6, SDS, disease duration, or monthly headache days (*p* > 0.05, uncorrected; Figure 7).

## 4 Discussion

This study systematically characterized abnormalities in the dynamic functional connectivity of the brains of young migraine patients using the HMM. By modeling resting-state fMRI data from 200 participants, we identified 11 stable and reproducible dynamic brain states and observed significant alterations in state occupancy, mean dwell time, transition patterns, and intra-state connectivity in the patient group. These findings offer novel insights into the neuropathological mechanisms underlying migraine.

### 4.1 State 9 connectivity abnormalities indicate disrupted multisensory integration and default network regulation

Edge-level analysis revealed significant functional connectivity alterations in State 9—a configuration characterized by coordinated activation across visual, sensorimotor, and salience-related regions. The primary abnormalities were located in DMN–SC cross-network connectivity, extending to DMN–VAN and visual network interactions. These disruptions carry substantial pathophysiological implications.

Abnormal coupling between the Default Mode Network (DMN) and sensorimotor cortex may be associated with differences in the interaction between internally oriented processes and sensory processing (Zhang et al., 2016). Under typical conditions, the DMN dominates during rest to support internal mentation, whereas the sensorimotor network processes peripheral sensory inputs (Menon, 2023). Effective cognitive functioning requires moderate segregation between these networks. In migraine patients, the altered DMN–SC connectivity observed in State 9 suggests that, upon entering this multisensory integration state, the normal boundary between internal and external attention systems is compromised. Such disruption may hinder the suppression of internal thoughts during sensory processing or allow peripheral sensory interference during internally oriented cognitive activities.

The abnormal DMN–VAN coupling further implies dysfunction in salience detection processes, whereby patients may attribute excessive significance to sensory stimuli that would normally be filtered out, triggering unnecessary attentional shifts and pain-related alerts (Zhao T. et al., 2021). The altered visual network connectivity aligns with the clinical feature of photophobia, suggesting reorganization within visual processing pathways themselves (Zhang et al., 2020). Importantly, these abnormalities were state-specific, appearing only in State 9 and not in other states, indicating that neuropathological changes in migraine are not static or globally distributed but manifest selectively under distinct dynamic configurations (Chang et al., 2021). MDT in State 9 was positively correlated with MIDAS scores, suggesting that prolonged engagement in this multisensory integration state may be associated with greater functional disability. Given that State 9 is characterized by concurrent activation of visual, sensorimotor, and salience networks together with reduced default mode and frontoparietal control network activity, extended dwell time in this state may reflect sustained sensory amplification and diminished top-down regulatory control. Such a configuration may increase the subjective burden of sensory input and reduce cognitive flexibility, thereby exacerbating migraine-related disability in daily life.

### 4.2 State 7 dynamic locking reflects sustained hypervigilance and sensory overmonitoring

The pronounced dynamic locking observed in State 7 warrants special consideration. This state is characterized by dominant activation of the Dorsal Attention Network (DNA), and migraine patients showed markedly increased fractional occupancy, prolonged mean dwell time, and enhanced self-transition probability. As the DAN governs top-down, goal-directed attentional control, its overactivation may reflect a state of persistent hypervigilance and exaggerated environmental monitoring in migraine patients (Bao et al., 2022).

Such a DAN-dominant locking pattern holds multiple mechanistic implications. First, it may represent a maladaptive adaptation to recurrent pain experiences: patients remain in a heightened vigilance state in an attempt to detect and avoid potential headache triggers—including bright lights, loud noises, or specific visual stimuli (Aoe et al., 2024; Black et al., 2021). However, prolonged attentional allocation toward external stimuli depletes cognitive resources and may underlie the attentional deficits and mental fatigue commonly reported in migraine (Chen et al., 2024). Second, sustained DAN overactivation may lower sensory thresholds, increasing susceptibility to external stimuli capable of provoking headache attacks, consistent with clinical observations of photophobia, phonophobia, and other sensory hypersensitivities (Han et al., 2023).

The contrast between State 7 (DAN-dominant) and State 9 (multisensory integration) locking is particularly revealing. Whereas State 7 reflects excessive orientation toward external stimuli, State 9 reflects impaired segregation between internal and external processing streams. Together, the co-locking of these dysfunctional states suggests that the migraine brain oscillates between two maladaptive modes: heightened external monitoring (State 7) and disorganized mixing of internal and external information (State 9). This dual dysfunction may help explain the variable and complex cognitive symptoms observed in migraine (Sedley et al., 2024).

### 4.3 Imbalanced distribution of dynamic states and network load alterations

Patients exhibited increased occupancy in States 6, 7, 9, and 5, alongside decreased occupancy in States 3, 4, 8, and 11. From a network load perspective, the states with increased occupancy were consistently characterized by elevated involvement of the visual system and dorsal attention network, whereas the states with reduced occupancy predominantly represented baseline sensorimotor configurations (Sedley et al., 2024).

This imbalance likely reflects maladaptive redistribution of neural resources in migraine. The elevated occupancy of visual–attention states suggests that patients' brains more frequently adopt configurations related to environmental monitoring and sensory processing, possibly as a compensatory strategy arising from repeated pain episodes (Wei et al., 2022). However, such overreliance may substantially burden cognitive resources. Conversely, diminished occupancy of baseline sensorimotor states may impair sensory gating processes, which are essential for maintaining perceptual thresholds and filtering non-noxious stimuli. Reduced engagement of these baseline configurations may render patients more vulnerable to everyday sensory triggers capable of inducing headache attacks.

## 5 Limitations

Several limitations warrant consideration. First, the cross-sectional design precludes causal inference; future longitudinal studies tracking brain states across the migraine cycle are necessary. Second, the sample consisted exclusively of young adults with MWoA during the interictal phase, which may constrain the generalizability of these findings to other age groups, migraine subtypes, or attack phases. Third, our statistical approach was primarily exploratory, and no a-priori power analysis was conducted to determine the sample size. This was due to the current lack of established effect size data for dynamic state parameters in interictal migraine. While our sample size is consistent with existing HMM studies in the field, the absence of prior power calculation—combined with the omission of additional covariates such as age, sex, head motion, and mood measures—means that residual confounding effects cannot be entirely ruled out. Specifically, the brain–clinical correlations identified in this study (e.g., the association between State 9 and MIDAS scores) did not undergo multiple-comparison correction across all states. Notably, the migraine group showed SDS scores nearing the threshold for depressive tendency. Because SDS was not modeled as a covariate, observed connectivity alterations may partly reflect affective symptoms rather than migraine-specific mechanisms. Similarly, anxiety symptoms and sleep disturbances were not systematically assessed (e.g., using GAD-7 or PSQI), which may have further influenced dynamic functional connectivity measures and confounded the results. Therefore, replication in larger, independent cohorts using pre-calculated sample sizes and stringent statistical corrections is essential to confirm these findings. Fourth, the HMM framework involves specific methodological assumptions. The Markov property assumes that state transitions depend only on the immediately preceding state, which may not capture longer-range temporal dependencies. Additionally, discretizing continuous neural activity into finite states may oversimplify the fluid nature of large-scale network interactions. Furthermore, our focus on network-level summaries limits mechanistic interpretability compared to detailed node- or edge-level analyses. Finally, pharmacological influences remain a potential confounder. Although we excluded participants receiving preventive treatment within 3 months prior to the study, the influence of acute medications (e.g., triptans or NSAIDs) used for symptomatic relief cannot be entirely ruled out. Due to the lack of exhaustive medication records, we were unable to systematically control for these effects. Future research utilizing medication-naive cohorts will be critical to isolating intrinsic pathological changes from treatment-related modulations. Despite these constraints, this study provides a valuable exploratory foundation for understanding the dynamic functional architecture of the migraine brain. Through addressing these limitations, future studies can further elucidate the complex neural mechanisms underlying this disorder.

## 6 Conclusions

Using Hidden Markov Models applied to resting-state fMRI data, this study identified 11 stable dynamic brain states in young migraine patients and revealed state-specific alterations. Patients exhibited prolonged dwelling in State 7 (a DAN-dominant configuration reflecting sustained hypervigilance) and State 9 (a multisensory integration state marked by disrupted DMN–SC and DMN–VAN connectivity), together with reduced occupancy of baseline sensorimotor states (States 3, 4, 8, 11). Mean dwell time in State 9 was correlated with MIDAS disability scores (*r* = 0.38, *p* < 0.05). This dual dysfunction—characterized by excessive external monitoring and disorganized internal–external integration—combined with enhanced transitions toward maladaptive states, reflects dysregulated neural resource allocation. These findings highlight the potential of HMM-derived dynamic states as biomarkers and therapeutic targets for migraine.

## Data Availability

The datasets presented in this article are not readily available because they contain sensitive clinical and neuroimaging information; the participants did not provide consent for public data sharing. Requests to access the datasets should be directed to the corresponding author.

## References

[B1] Al-Hashel J. Y. Alroughani R. Gad K. Al-Sarraf L. Ahmed S. F. (2022). Risk factors of white matter hyperintensities in migraine patients. BMC Neurol. 22:159. doi: 10.1186/s12883-022-02680-8 35488255 PMC9052543

[B2] Amiri P. Kazeminasab S. Nejadghaderi S. A. Mohammadinasab R. Pourfathi H. Araj-Khodaei M. . (2021). Migraine: a review on its history, global epidemiology, risk factors, and comorbidities. Front. Neurol. 12:800605. doi: 10.3389/fneur.2021.800605 35281991 PMC8904749

[B3] Aoe T. Kawanaka R. Ohsone F. Hara A. Yokokawa T. (2024). Functional connectivity associated with attention networks differs among subgroups of fibromyalgia patients: an observational case-control study. Sci. Rep. 14:10197. doi: 10.1038/s41598-024-60993-9 38702506 PMC11068894

[B4] Bao S. Qiao M. Lu Y. Jiang Y. (2022). Neuroimaging mechanism of cognitive behavioral therapy in pain management. Pain Res. Manag. 2022:6266619. doi: 10.1155/2022/6266619 35154551 PMC8828323

[B5] Black S. R. King J. B. Mahan M. A. Anderson J. Butson C. R. (2021). Functional hyperconnectivity and task-based activity changes associated with neuropathic pain after spinal cord injury: a pilot study. Front. Neurol. 12:613630. doi: 10.3389/fneur.2021.613630 34177753 PMC8222514

[B6] Chang C. Yang C. Yang C. Shih P. Wang S. (2021). Evidence of potential mechanisms of acupuncture from functional MRI data for migraine prophylaxis. Curr. Pain Headache Rep. 25:49. doi: 10.1007/s11916-021-00961-4 34036477

[B7] Chen Y. Xie S. Zhang L. Li D. Su H. Wang R. . (2024). Attentional network deficits in patients with migraine: behavioral and electrophysiological evidence. J. Headache Pain 25:195. doi: 10.1186/s10194-024-01905-0 39528969 PMC11552239

[B8] Cloet F. Gueyraud G. Lerebours F. Munio M. Larrue V. Gollion C. (2024). Stroke due to small-vessel disease and migraine: a case-control study of a young adult with ischemic stroke population. Cephalalgia 44:3331024241282015. doi: 10.1177/03331024241282015 39512081

[B9] Dosenbach N. U. F. Nardos B. Cohen A. L. Fair D. A. Power J. D. Church J. A. . (2010). Prediction of individual brain maturity using fMRI. Science 329, 1358–1361. doi: 10.1126/science.1194144 20829489 PMC3135376

[B10] Gao Q. Xu F. Jiang C. Chen Z. Chen H. Liao H. . (2016). Decreased functional connectivity density in pain-related brain regions of female migraine patients without aura. Brain Res. 1632, 73–81. doi: 10.1016/j.brainres.2015.12.007 26688226

[B11] Geng L. Feng Q. Wang X. Sun J. Tang S. Jia H. . (2025). Depression links to unstable resting-state brain dynamics: insights from hidden markov models and functional network variability. Psychol. Med. 55:e200. doi: 10.1017/S0033291725101001 40671332 PMC12315643

[B12] Han L. Lu J. Chen C. Ke J. Zhao H. (2023). Altered functional connectivity within and between resting-state networks in patients with vestibular migraine. Neuroradiology 65, 591–598. doi: 10.1007/s00234-022-03102-9 36520172

[B13] Hu S. Hao Z. Li M. Zhao M. Wen J. Gao Y. . (2023). Resting-state abnormalities in functional connectivity of the default mode network in migraine: a meta-analysis. Front. Neurosci. 17:1136790. doi: 10.3389/fnins.2023.1136790 36937687 PMC10014826

[B14] Huskisson E. C. (1974). Measurement of pain. Lancet 2, 1127–1131. doi: 10.1016/S0140-6736(74)90884-8 4139420

[B15] Hussain S. Langley J. Seitz A. R. Hu X. P. Peters M. A. K. (2023). A novel hidden markov approach to studying dynamic functional connectivity states in human neuroimaging. Brain Connect. 13, 154–163. doi: 10.1089/brain.2022.0031 36367193 PMC10079241

[B16] Ingram B. T. Mayhew S. D. Bagshaw A. P. (2024). Brain state dynamics differ between eyes open and eyes closed rest. Hum. Brain Mapp. 45:*e*26746. doi: 10.1002/hbm.26746 38989618 PMC11237880

[B17] Jiang F. Jin H. Gao Y. Xie X. Cummings J. Raj A. . (2022). Time-varying dynamic network model for dynamic resting state functional connectivity in fMRI and MEG imaging. Neuroimage 254:119131. doi: 10.1016/j.neuroimage.2022.119131 35337963 PMC9942947

[B18] Kosinski M. Bayliss M. S. Bjorner J. B. Ware J. E. J. Garber W. H. Batenhorst A. . (2003). A six-item short-form survey for measuring headache impact: the HIT-6. Qual Life Res. 12, 963–974. doi: 10.1023/A:1026119331193 14651415

[B19] Lee M. J. Park B. Cho S. Park H. Kim S. Chung C. (2019). Dynamic functional connectivity of the migraine brain: a resting-state functional magnetic resonance imaging study. Pain 160, 2776–2786. doi: 10.1097/j.pain.0000000000001676 31408050

[B20] Menon V. (2023). 20 years of the default mode network: A review and synthesis. Neuron 111, 2469–2487. doi: 10.1016/j.neuron.2023.04.023 37167968 PMC10524518

[B21] Olesen J. (2018). International classification of headache disorders. Lancet Neurol. 17, 396–397. doi: 10.1016/S1474-4422(18)30085-1 29550365

[B22] Savva A. D. Mitsis G. D. Matsopoulos G. K. (2019). Assessment of dynamic functional connectivity in resting-state fMRI using the sliding window technique. Brain Behav. 9:e01255. doi: 10.1002/brb3.1255 30884215 PMC6456784

[B23] Schwedt T. J. Chiang C. Chong C. D. Dodick D. W. (2015). Functional MRI of migraine. Lancet Neurol. 14, 81–91. doi: 10.1016/S1474-4422(14)70193-0 25496899 PMC11318354

[B24] Sedley W. Kumar S. Jones S. Levy A. Friston K. Griffiths T. . (2024). Migraine as an allostatic reset triggered by unresolved interoceptive prediction errors. Neurosci. Biobehav. Rev. 157:105536. doi: 10.1016/j.neubiorev.2024.105536 38185265

[B25] Skorobogatykh K. Van Hoogstraten W. S. Degan D. Prischepa A. Savitskaya A. Ileen B. M. . (2019). Functional connectivity studies in migraine: what have we learned? J Headache Pain 20:108. doi: 10.1186/s10194-019-1047-3 31747874 PMC6868768

[B26] Steiner T. J. Stovner L. J. Jensen R. Uluduz D. Katsarava Z. (2020). Migraine remains second among the world's causes of disability, and first among young women: findings from GBD2019. J. Headache Pain 21:137. doi: 10.1186/s10194-020-01208-0 33267788 PMC7708887

[B27] Stewart W. F. Lipton R. B. Dowson A. J. Sawyer J. (2001). Development and testing of the migraine disability assessment (MIDAS) Questionnaire to assess headache-related disability. Neurology 56, S20–S28. doi: 10.1212/WNL.56.suppl_1.S20 11294956

[B28] Tessitore A. Russo A. Giordano A. Conte F. Corbo D. De Stefano M. . (2013). Disrupted default mode network connectivity in migraine without aura. J. Headache Pain 14:89. doi: 10.1186/1129-2377-14-89 24207164 PMC3832236

[B29] Vergara V. M. Abrol A. Calhoun V. D. (2019). An average sliding window correlation method for dynamic functional connectivity. Hum. Brain Mapp. 40, 2089–2103. doi: 10.1002/hbm.24509 30659699 PMC6865616

[B30] Wei H. Tian T. Zhou G. Wang J. Guo X. Chen Y. . (2022). Disrupted dynamic functional connectivity of the visual network in episodic patients with migraine without aura. Neural Plast. 2022:9941832. doi: 10.1155/2022/9941832 35035474 PMC8754605

[B31] Zacher H. Dirkers B. T. Korek S. Hughes B. (2017). Age-differential effects of job characteristics on job attraction: a policy-capturing study. Front. Psychol. 8:1124. doi: 10.3389/fpsyg.2017.01124 28713322 PMC5491976

[B32] Zhang D. Huang X. Su W. Chen Y. Wang P. Mao C. . (2020). Altered lateral geniculate nucleus functional connectivity in migraine without aura: a resting-state functional MRI study. J. Headache Pain 21:17. doi: 10.1186/s10194-020-01086-6 32066379 PMC7025412

[B33] Zhang J. Su J. Wang M. Zhao Y. Yao Q. Zhang Q. . (2016). Increased default mode network connectivity and increased regional homogeneity in migraineurs without aura. J. Headache Pain 17:98. doi: 10.1186/s10194-016-0692-z 27771875 PMC5075323

[B34] Zhao Q. Liu R. Zhou J. Dong Z. Chen Y. (2021). Prevalence and grade of RLS in migraine: A prospective study of 251 migraineurs by synchronous test of c-TTE and c-TCD. Medicine 100:e24175. doi: 10.1097/MD.0000000000024175 33530208 PMC7850732

[B35] Zhao T. Pei L. Ning H. Guo J. Song Y. Zhou J. . (2021). Networks are associated with acupuncture treatment in patients with diarrhea-predominant irritable bowel syndrome: a resting-state imaging study. Front. Hum. Neurosci. 15:736512. doi: 10.3389/fnhum.2021.736512 34720908 PMC8551866

[B36] Zhou Y. Gong L. Yang Y. Tan L. Ruan L. Chen X. . (2023). Spatio-temporal dynamics of resting-state brain networks are associated with migraine disability. J. Headache Pain 24:13. doi: 10.1186/s10194-023-01551-y 36800935 PMC9940435

[B37] Zou Y. Tang W. Qiao X. Li J. (2021). Aberrant modulations of static functional connectivity and dynamic functional network connectivity in chronic migraine. Quant. Imaging Med. Surg. 11, 2253–2264. doi: 10.21037/qims-20-588 34079699 PMC8107335

[B38] Zung W. W. (1965). A self-rating depression scale. Arch. Gen. Psychiatry 12, 63–70. doi: 10.1001/archpsyc.1965.01720310065008 14221692

