# Supplementary figures and images for: State-specific disruptions of dynamic functional connectivity in young migraine without aura: a hidden Markov model approach
Source: Front Neurosci. 2026 Mar 18;20:1756997. doi: 10.3389/fnins.2026.1756997 (PMC13038879; doi:10.3389/fnins.2026.1756997)

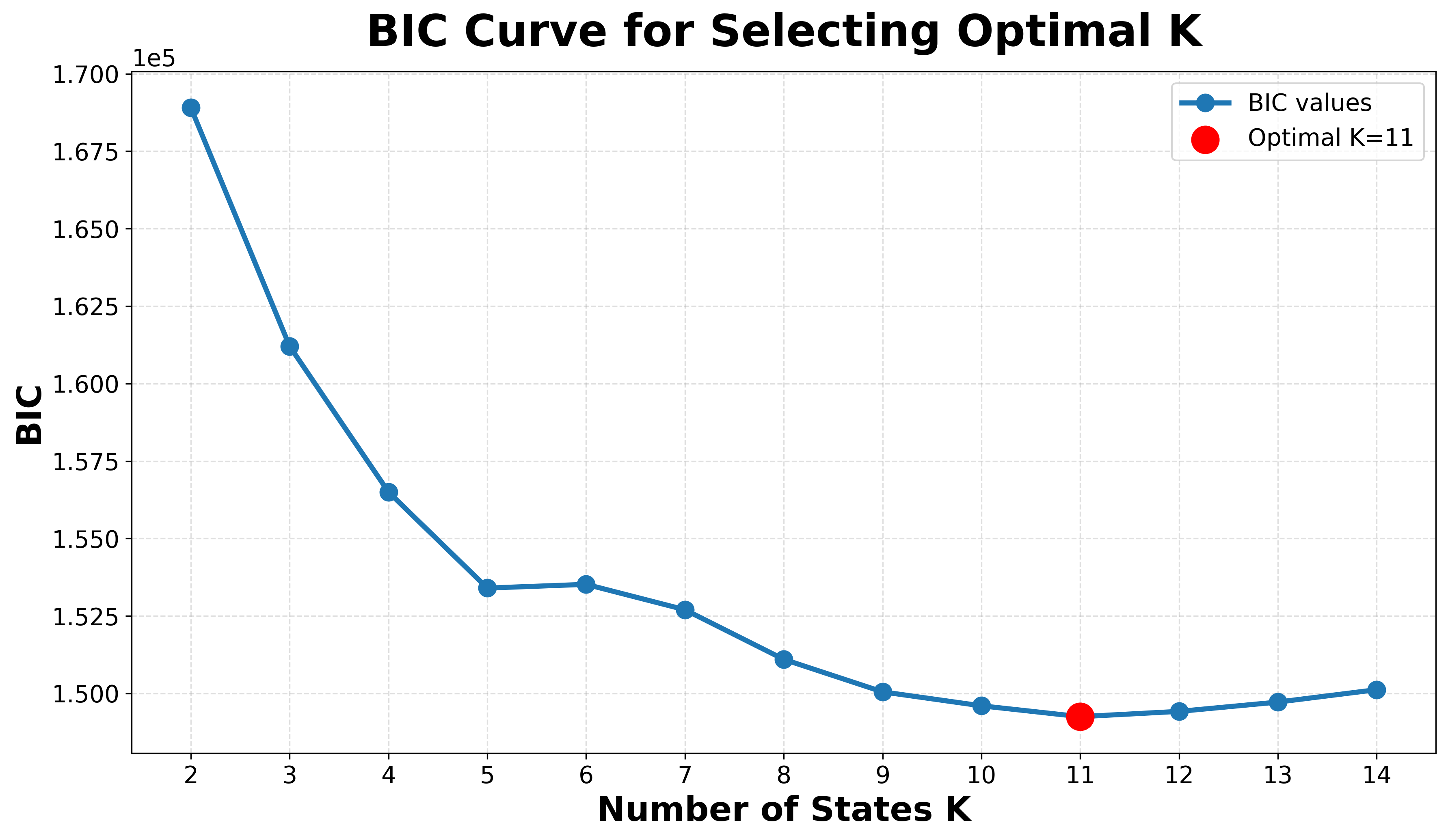

Supplement: Supplementary file 1 [file Image_1.png]

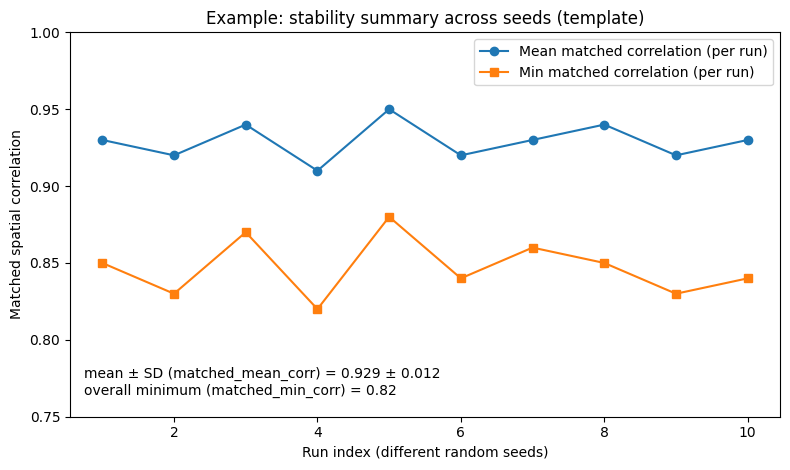

Supplement: Supplementary file 2 [file Image_2.png]
